# Evolution of Secondary α Phase during Aging Treatment in Novel near β Ti-6Mo-5V-3Al-2Fe Alloy

**DOI:** 10.3390/ma11112283

**Published:** 2018-11-15

**Authors:** Haoyu Zhang, Chuan Wang, Siqian Zhang, Ge Zhou, Lijia Chen

**Affiliations:** 1 School of Materials Science and Engineering, Shenyang University of Technology, Shenyang 110870, China; sqzhang@alum.imr.ac.cn (S.Z.); zhouge1985@sina.com (G.Z.); chenlijia@sut.edu.cn (L.C.); 2 College of Light Industry, Liaoning University, Shenyang 110036, China; wangc1101@126.com

**Keywords:** near β titanium alloy, secondary α phase, microstructural evolution, aging treatment

## Abstract

Evolution of secondary α phase during aging treatment of a novel near β titanium alloy Ti-6Mo-5V-3Al-2Fe(wt.%) was studied by OM, SEM, and TEM. Results indicated that size and distribution of secondary α phase were strongly affected by aging temperature and time. Athermal ω phase formed after super-transus solution treatment followed by water quenching, and promoted nucleation of needle-like intragranular α in subsequent aging process. When aged at 480 °C, fine scaled intragranular α with small inter-particle spacing precipitated within β grains and high ultimate tensile strength above 1500 MPa was achieved. When the aging temperature increased, the size and inter-particle spacing of intragranular α increased and made the strength reduce, but the ductility got improved. When aging temperature reached as high as 600 °C, ω phase disappeared and intragranular α coarsened obviously, resulting in serious decrease of strength. While mutually parallel Widmanstätten α laths formed at the vicinity of β grain boundaries and grew into the internal area of β grains, and significant improvement of ductility was achieved. As the aging time increased from 4 h to 16 h at 600 °C, the intragranular α grew slightly and brought about minor change of mechanical properties.

## 1. Introduction

Near β titanium alloys are considered as excellent alternatives to replace steels for structural applications in the aerospace industry due to their high specific strength, good corrosion resistance, and fatigue crack growth resistance [1,2,3,4,5]. For instance, the Ti-5Al-5Mo-5V-3Cr (Ti-5553) alloy and the Ti-10V-2Fe-3Al (Ti-1023) alloy have been applied on landing gear forgings for Airbus-350 and Boeing 777, respectively [6,7]. The strength and ductility of near β titanium alloys are strongly influenced by size, morphology, and distribution of secondary α phase formed after aging treatment [8]. In general, three types of secondary α phase, in terms of precipitation sites, are commonly formed in near β titanium alloys, i.e., grain boundary α that precipitates on β grain boundaries, Widmanstätten α ($\alpha_{\mathrm{WGB}}$) that precipitates at vicinities of β grain boundaries and intragranular α that precipitates within β grains [9]. Near β titanium alloys possess extremely high strength due to precipitation of finely dispersed intragranular α precipitates [10]. However, the improvement of strength is usually accompanied by a decrease in ductility, resulting from unexpected microstructural characteristics, such as grain boundary α [11,12]. Generally, an excellent combination of strength and ductility is strongly dependent on size, morphology, and distribution of secondary α phase [13].

In recent years, significant efforts have been made to investigate the microstructure evolution of α phase during aging treatment in order to meet the requirement for high strength and acceptable ductility of near β titanium alloys. Ren et al. [14] investigated the effect of aging temperature on microstructure and mechanical properties of a novel near β titanium alloy Ti-5Al-3Mo-3V-2Cr-2Zr-1Nb-1Fe and assumed that intragranular α precipitates coarsened due to the increase of aging temperature. He et al. [15] studied the microstructural evolution of α precipitates during aging process of a commercial β titanium alloy β-CEZ (Ti-5Al-2Sn-4Zr-4Mo-2Cr-1Fe) using high-resolution transmission electron microscopy, and proposed that fine needle-like intragranular α with 5–10 nm in width formed as a result of ω-assisted nucleation. Ahmed et al. [16] reported various sizes and volume fractions of secondary α phase during aged at 500–725 °C in a commercial near β titanium alloy Ti-10V-2Fe-3Al. Hua et al. [17] reported that the $\alpha_{\mathrm{WGB}}$ appeared after being solution treated at 900 °C for 0.5 h followed by aging at 700 °C for 5 min in β titanium alloy Ti-7Mo-3Nb-3Cr-3Al. However, the effect of $\alpha_{\mathrm{WGB}}$ on mechanical properties was not discussed in their work.

Ti-6Mo-5V-3Al-2Fe (wt.%) alloy is a newly designed near β titanium alloy according to the molybdenum equivalence ([Mo]_eq_) and d-electron theory. As a newly designed alloy, the effect of aging treatment on microstructure and mechanical properties of the alloy had not been investigated by far. The present work is to investigate microstructural characteristics of secondary α phase in the alloy, in terms of aging treatment at temperature from 480 °C to 600 °C for a wide time range from 4 h to 16 h. The objective is to understand the effect of aging temperature and time on microstructural evolution. In addition, the mechanical properties of the aged alloys were evaluated by tensile test to study the role of microstructural evolution of secondary α phase in affecting mechanical properties. The results of present work could provide meaningful references for improving mechanical properties of Ti-6Mo-5V-3Al-2Fe alloy, which is expected to show potential for high-strength structural applications in the aerospace industry.

## 2. Materials and Methods

### 2.1. Alloy Design

**Figure 1:**
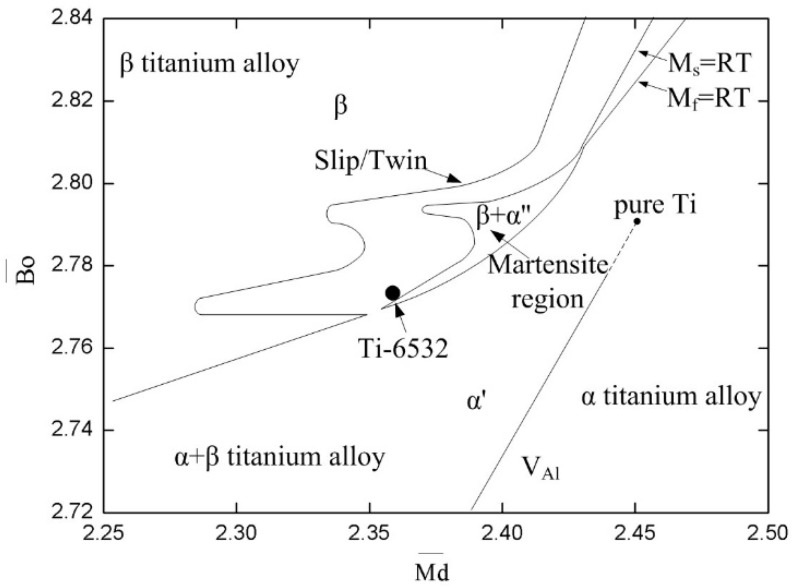
The position of the alloy in $\bar{Bo}$–$\bar{Md}$ diagram.

According to the d-electron theory, suitable stability of β phase after quenching can be achieved by tailoring the $\bar{Bo}$ and $\bar{Md}$ parameters, in terms of the Morinaga’s phase stability $\bar{Bo}$−$\bar{Md}$ diagram [18]. Many β titanium alloys with excellent mechanical properties have been designed based on the d-electron theory, such as Ti-8.5Cr-1.5Sn (wt.%) alloy with extra high strain-hardening rate and Ti-6Cr-4Mo-2Al-2Sn-1Zr (wt.%) alloy with high product of strength and elongation of 42.6 GPa% [19,20]. Ti-6Mo-5V-3Al-2Fe alloy was designed, based on the d-electron theory. After deliberating *Bo* and *Md* parameters of every element, the isomorphous Mo and V and eutectoid Fe were used for stabilizing β phase. The powerful β-stabilizer Fe and relatively high Mo contents were added in order to reduce the expensive V cost. Besides, the α-stabilizer element Al was added to reduce the stability of β phase, in order to promote aging precipitation. In addition, the contents of Mo, V, Al and Fe were carefully optimized for tailoring the alloy’s position in the $\bar{Bo}$−$\bar{Md}$ diagram. With regard to the designed alloy, values of $\bar{Bo}$ and $\bar{Md}$ are about 2.777 and 2.366, respectively. According to $\bar{Bo}$−$\bar{Md}$ diagram, the position of designed alloy was located outside the martensite region and closed to the border of β titanium alloy region as shown in Figure 1. In addition, corresponding position is located closely to the conventional high strength metastable/near β titanium alloys, such as Ti-1023, BT22 and β-21S [14,21].

Conventionally, [Mo]_eq_ was used to design titanium alloys. For β titanium alloys, it is known that a suitable value of [Mo]_eq_ is required to achieved high strength, which is normally around 8–14 [22]. The [Mo]_eq_ is defined as the sum of the weighted averages of elements present in a titanium alloy and usually used to quantify the stability of β phase. The equations of [Mo]_eq_ is given as:

[Mo]_eq_ = 1.0[Mo] + 0.67[V] + 0.44[W] + 0.28[Nb] + 0.22[Ta] + 2.9[Fe] + 1.6[Cr] + 1.54[Mn] + 1.25[Ni] − 1.0[Al] (1)

According to the Equation (1), the [Mo]_eq_ of designed alloy is 12.15, which belongs to near β titanium alloy and falls into the high-strength range around 8–14. According to the d-electron theory and [Mo]_eq_, it can be predicted that low stability of β phase after quenching, which is beneficial to aging precipitation can be obtained and high strength, can be expected.

### 2.2. Alloy Preparation

**Figure 2:**
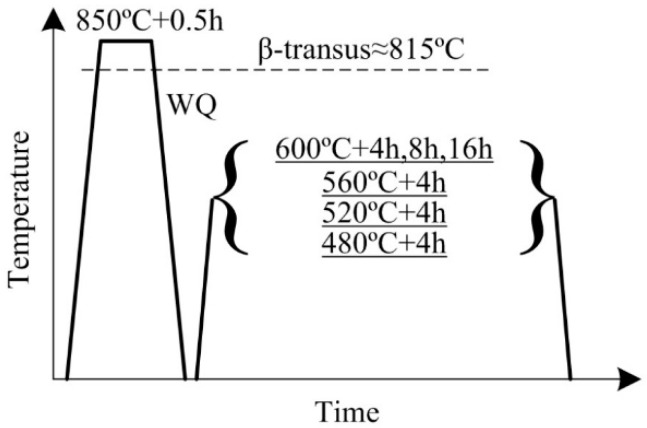
Schematic illustration of the heat treatment.

Sponge Ti, pure Mo, pure Al, pure Fe and V-Al master alloy were used to prepare the alloy ingot. Raw materials were melted twice by vacuum arc remelting to ensure chemical homogeneity. The β-transus temperature of the alloy verified by metallographic method is 815 °C ± 5 °C. The ingot with 120 mm in diameter and 200 mm in length was first forged to plate with 100mm in width and 30 mm in thickness. The plate was soaked in β field and then through multi-pass hot rolled into plate with 6 mm in thickness followed by air cooling. The as-rolled alloy was super-transus solution-treated followed by water cooling. Then, the as-quenched alloy was aged at various temperatures and times, all ended by air cooling. Detailed parameters of the heat treatment are shown in Figure 2.

Two kinds of modified kroll’s reagent (10 mL HF + 30 mL HNO_3_ + 50 mL H_2_O and 10 mL HF + 30 mL HNO_3_ + 80 mL H_2_O) were adopted to reveal the microstructures of the quenched and aged alloys. The microstructures were characterized by optical microscope (OM, Zeiss AXIO Observer.A1m, Carl Zeiss AG, Jena, Germany), scanning electron microscopy (SEM, Hitachi Su8010, Hitachi, Tokyo, Japan)) and transmission electron microscopy (TEM, JEOL Jem-2100, JEOL, Tokyo, Japan)). TEM samples were cut in the center of the aged specimens by electric sparking. Then, TEM samples were manually ground to 50 μm and twin-jet electropolished in a solution (6 vol.% perchloric acid, 35 vol.% *n*-butyl alcohol and 59 vol.% methanol) at −20 °C. Mechanical properties were evaluated by tensile test at room temperature at a strain rate of 10^−3^ s^−1^ on MTS landmark 370.10 servohydraulic test system.

**Figure 3:**
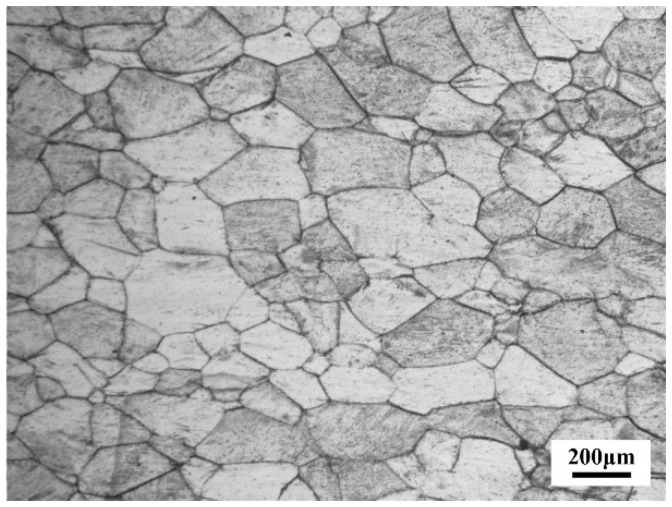
Microstructure of the as-quenched alloy.

After super-transus solution-treated at 850 °C for 0.5 h followed by water quenching, the alloy exhibited single phase microstructure of large equiaxed β grains (grain scale is ~200 μm), as shown in Figure 3.

## 3. Results

### 3.1. Secondary α Phase after Being Aged at Various Temperatures

**Figure 4:**
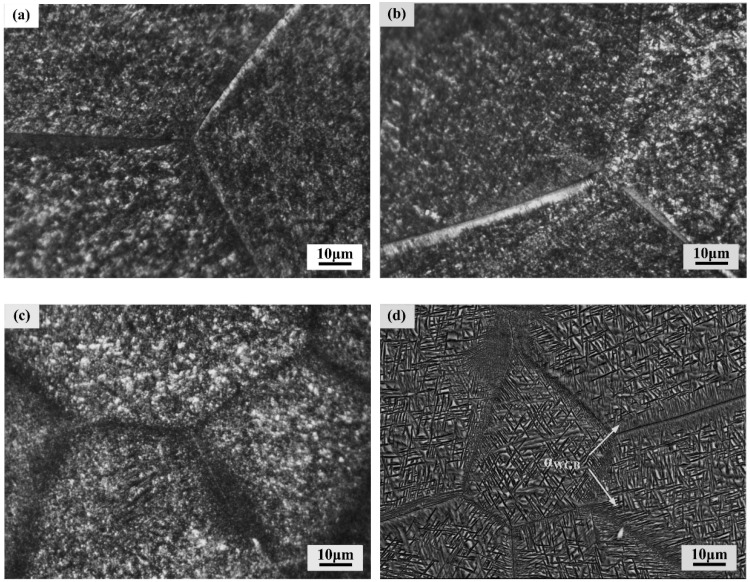
Microstructure of the alloys aged at (**a**) 480 °C; (**b**) 520 °C; (**c**) 560 °C; (**d**) 600 °C for 4 h.

**Figure 5:**
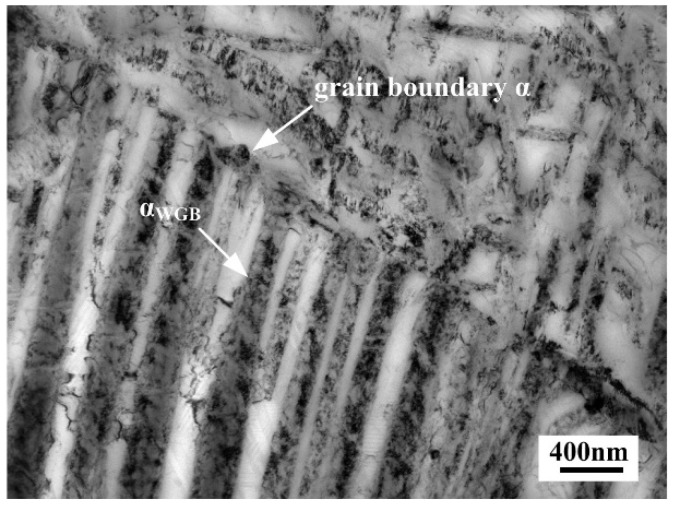
TEM micrograph of $\alpha_{\mathrm{WGB}}$ near β grain boundaries in the alloy aged at 600 °C for 4 h.

Figure 4 shows the microstructures of the alloy aged at 480–600 °C for 4 h. Microstructures of the alloy aged at 480 °C, 520 °C, 560 °C and 600 °C all consisted of α precipitates uniformly distributed within β grains. In the alloy aged at 480 °C, 520 °C and 560 °C, intragranular α was too fine to be identified by OM micrographs. Whereas, when aging temperature reached 600 °C, the needle-like morphology of intragranular α could be clearly found due to noticeable coarsening of intragranular α. In addition, when aged at 600 °C, mutually parallel laths of α phase formed along the β grain boundaries. Similar morphology of such α phase has been reported by previous literature, and the parallel colony that observed as Widmanstätten laths near prior β grain boundaries can be designated as $\alpha_{\mathrm{WGB}}$ [23] Hence further observation of the $\alpha_{\mathrm{WGB}}$ was carried out on TEM, as shown in Figure 5. It could be clearly observed that mutually parallel $\alpha_{\mathrm{WGB}}$ precipitated on continuous grain boundary α and grew into the internal areas of β grains.

**Figure 6:**
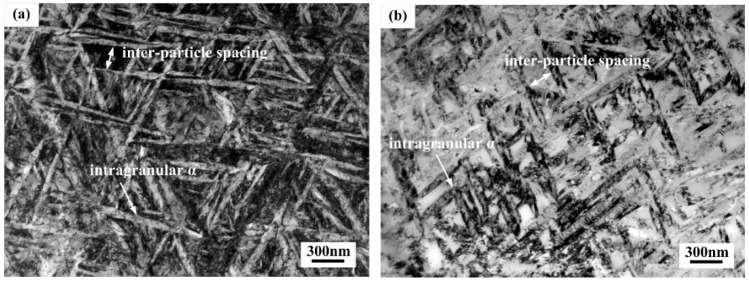

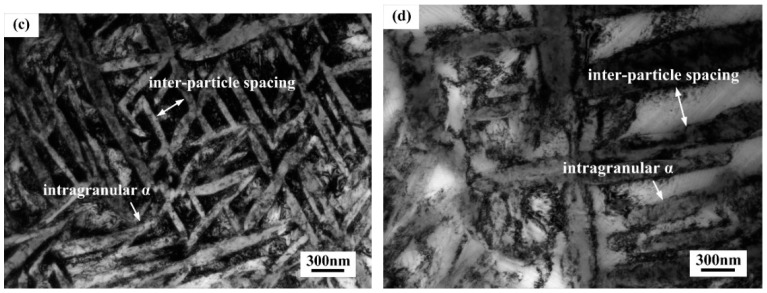
TEM micrographs of intragranular α in the alloy aged at (**a**) 480 °C; (**b**) 520 °C; (**c**) 560 °C (**d**) 600 °C for 4 h.

Observations of intragranular α were carried out on TEM, in order to further understand the evolution of intragranular α that affected by aging temperature. Figure 6 shows TEM micrographs of the alloy aged at 480–600 °C for 4 h, in which denser dispersion of intragranular α with high aspect ratios are presented. The increase of aging temperature induced the growth of intragranular α, while coarser α precipitates gave rise to broader inter-particle spacing. Meanwhile, it is worthy of notice that coarsening of intragranular α precipitated at 600 °C was obviously greater than that at lower aging temperatures.

### 3.2. Secondary α Phase after Being Aged for Different Time

**Figure 7:**
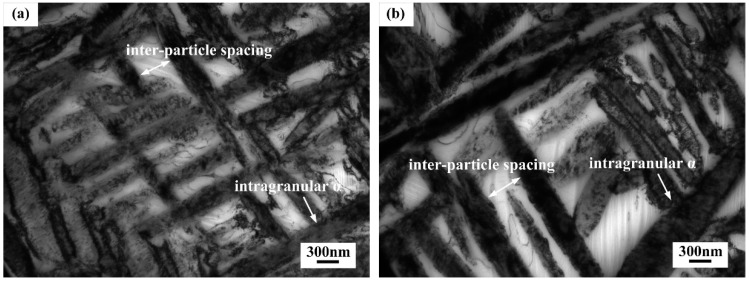
TEM micrographs of intragranular α phase in the alloy aged at 600 °C for (**a**) 8h (**b**) 16 h.

Figure 7 shows the TEM micrographs of intragranular α in the alloy aged at 600 °C for 8 h and 16 h. In comparison with Figure 6d, it seemed that intragranular α kept coarsening while its inter-particle spacing increased as the result of prolonged aging at 600 °C. However, the growth of intragranular α and their inter-particle spacing were not very obviously observed.

## 4. Discussion

### 4.1. Evolution of Secondary α Phase within β Grains

**Figure 8:**
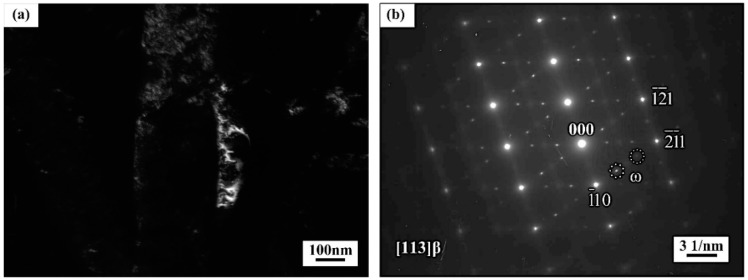
(**a**) Dark-field TEM micrograph of the as-quenched alloy; (**b**) corresponding electron diffraction patterns of β matrix.

Averaged width and inter-particle spacing of intragranular α was quantitatively calculated, as shown in Table 1. Intragranular α formed after aging at 480 °C exhibited smaller size (~67 nm in width) and inter-particle spacing (~79 nm) than that being aged at higher temperatures ranging from 480 °C to 600 °C. The precipitation of more and fine α phase at lower aging temperature from 480 °C to 600 °C can be attributed to the two following reasons. On the one hand, for near β titanium alloys, some kinds of precursors can form before aging treatment and assist the nucleation of secondary α phase during aging treatment. One of the possible precursors is the ω phase, which can form after solution treatment followed by water quenching [24]. Figure 8a,b shows the dark-field TEM image and selected-area diffraction patterns from β matrix of the as-quenched alloy, respectively. The selected-area diffraction patterns were indexed according to [113] zone axis of β phase. Additional quite weaker spots were visible at the 1/3 (121) and 2/3 (121) positions of the β reflection. These additional spots were referred to as the ω phase in β titanium alloy Ti-2Al-9.2Mo-2Fe [25]. The presence of these additional spots was direct evidence of ω precipitates within the β matrix of the as-quenched alloy. Such ω phase formed during quenching was called athermal ω phase, which appeared in many near β titanium alloy [15,26]. Lots of studies have reported that ω phase could assist the nucleation of α phase during aging treatment in near β titanium alloy [27,28,29]. The work of Dong et al. has demonstrated that the ω phase serves as the preferred site for nucleation of α phase and ω/β interface provides a profitable channel for the evolution of α phase during the heating process [30]. Therefore, it can be assumed that the ω phase formed after quenching could induce the nucleation of intragranular α and promote the uniform distribution during aging treatment in the current alloy. On the other hand, for near β titanium alloys, the transformation driving force will be the dominating factor for precipitation [31]. Lower aging temperature provided a higher undercooling degree and brought about larger transformation driving force and higher nucleation rate. Furthermore, the elements diffusion rate decreased with the aging temperature, resulting in slower growing rate of intragranular α. Therefore, the ω-assisted nucleation, high nucleation rate and slow growth rate of intragranular α all contributed to the formation of finely and dispersed intragranular α at 480 °C.

The higher aging temperature could provide larger driving force for the intragranular α growth but reduced the nucleation driving force due to the lower undercooling, so that coarse intragranular α phase precipitated accordingly. The precipitation of α phase formed a region with enriched β-stabilizer near the precipitates due to its low tolerance of β-stabilizers. In such region, the precipitation was difficult due to its high stability. So coarser intragranular α precipitates gave rise to broader β-stabilizer enriched region i.e., the inter-particle spacing of intragranular α. As a result, averaged width and inter-particle spacing of intragranular αincreased with the aging temperature.

**Figure 9:**
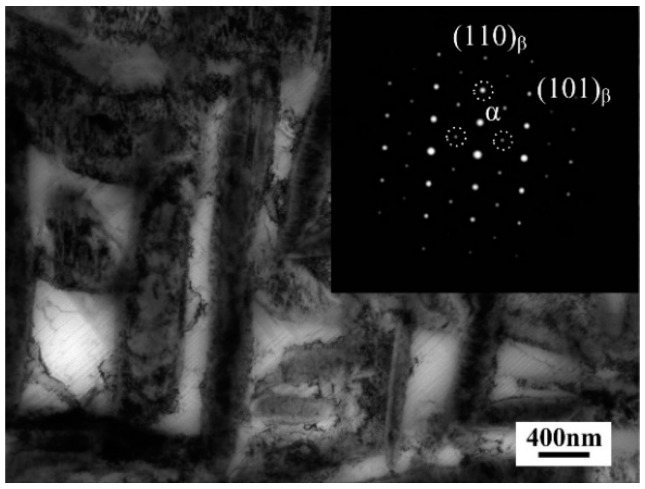
TEM micrograph of the alloy aged at 600 °C for 4 h and corresponding electron diffraction pattern.

It is worthy of notice that intragranular α phase grew noticeably after aging at 600 °C than that aged at other temperatures. This trend can be attributed to the disappearance of ω phase. Figure 9 shows the TEM micrograph of the alloy aged at 600 °C for 4 h. The inset of corresponding electron diffraction pattern indicated that there was no spots of ω phase, suggesting that ω phase disappeared after aged at 600 °C for sufficient time. The similar phenomenon has occurred in other β titanium alloys, such as Ti-7Mo-3Nb-3Cr-3Al alloy [30] and Ti-2Al-9.2Mo-2Fe alloy [25]. Their TEM observations indicated that the ω phase disappeared after being heated to 600 °C due to its metastable feature at high temperature. The disappearance of ω phase led to decreased concentration of β-stabilizers in residual β phase owing to its low tolerance of β-stabilizers [32]. Therefore, when aging temperature reached as high as 600 °C, as a result of high growth rate and low nucleation rate of α phase caused by high temperature, low stability of residual β phase tended to form coarser intragranular α.

Furthermore, according to Table 1, when aged at 600 °C, the increase of aging time resulted in slight growth of intragranular α. This trend can be ascribed to fast aging response at high temperature. At the beginning of aging treatment, the concentration of β-stabilizers in β matrix was low so that intragranular α phase grew considerably fast. Due to the high diffusion rate caused by high temperature, the intragranular α had grown sufficiently and surrounded by the region riched of β-stabilizer, consequently, the growth rate of intragranular α declined to the equilibrium state.

### 4.2. Evolution of Secondary α Phase near β Grain Boundary

When the heating temperature entering the α + β regime, the continuous grain boundary α formed preferentially due to the supply of nucleation sites by β grain boundaries consisting of large amount of defects [33]. Meanwhile, the alloying elements of β-stabilizer, particularly Mo, which is a slow-diffusing element, got enriched at the vicinity of β phase [34]. Such region was so chemically stable so that precipitation could not happen easily. With the increase of aging temperature, high temperature would enhance the diffusion of β-stabilizer, resulting in decrease of stability of the region near the β grain boundaries. When temperature reached critical value, the precipitation could get sufficient driving force. However, the distribution of precipitates near the β grain boundaries was different from that within the β grains. For the precipitation occurring within β grains, dislocations and precursors could exist as the nucleation sites, which promote the heterogeneous nucleation and made the secondary α phase irregularly distribute [35]. For the precipitation occurring near β grain boundaries, the nucleation sites would be mainly provided by continuous grain boundary α so that the precipitates distributed homogeneously. As a result, when aging temperature reached as high as 600 °C, the mutually parallel $\alpha_{\mathrm{WGB}}$ formed on grain boundaries and grew into β grains interior.

### 4.3. Effect of Secondary α Phase on Tensile Properties

**Figure 10:**
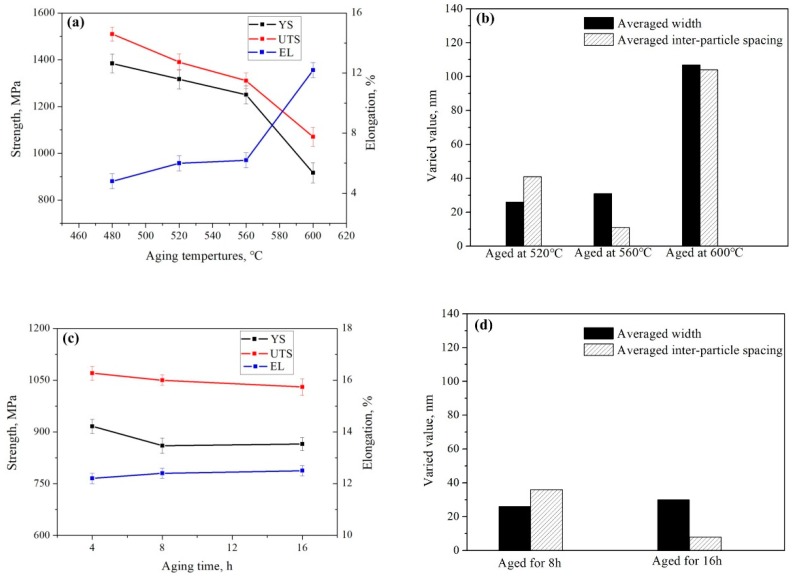
(**a**) Tensile properties of the alloy aged at 480 °C, 520 °C, 560 °C and 600 °C for 4 h; (**b**) varied values of the averaged width and inter-particle spacing of intragranular α in the alloy aged 520 °C, 560 °C and 600 °C for 4 h; (**c**) tensile properties of the alloy aged at 600 °C for 4 h, 8 h and 16 h; (**d**) varied values of average width and inter-particle spacing of intragranular α in the alloy aged at 600 °C for 8 h and 16 h.

In an attempt to better understand the effect of secondary α phase on the mechanical properties, tensile test was performed. Experimental results of tensile test, such as yield strength (YS) ultimate tensile strength (UTS) and elongation (EL), were shown in Figure 10a,c. Varied value of averaged width and inter-particle spacing of intragranular α after aging treatment were shown in Figure 10b,d.

Compared with the alloy aged at higher temperatures, smaller size and inter-particle spacing of intragranular α formed in the alloy aged at 480 °C provided more α/β interfaces, which acted as effective dislocation barriers [14]. In addition, the ω phase dispersed in β matrix strengthened the alloy effectively. Hence, as shown in Figure 10a, the alloy aged at 480 °C exhibited an excellent strength of 1510 MPa in UTS. However, the grain boundary α was relatively softer compared with the aging-hardened β matrix and had negative effect on ductility, because of its poor crack propagation resistance. In addition, the occurrence of ω phase could lead to a significant decrease in ductility due to its considerable brittleness [36]. Therefore, as shown in Figure 10a, the alloy aged at 480 °C exhibited a poor ductility of 4.2% in EL.

The influence of α phase on the yield strength of β titanium alloy can be expressed by the following equation [37]:

(2) $$\sigma_{y}=\frac{K_{p}}{l_{p}}+\frac{K_{s}}{l_{s}}$$

where, *σ_y_* is the yield strength of β titanium alloy, *l_p_* is the inter-particle spacing of primary α phase, *l_s_* is the inter-particle spacing of secondary α phase, *K_p_* and *K_s_* are constants with respect to Taylor factor. As indicated in Equation (2), the alloy strength decreased with the increase of inter-particle spacing among secondary α phases. According to Figure 10a,b, when aging temperature increased from 480 °C to 560 °C, the minor decrease of strength could be ascribed to the minor increase of inter-particle spacing of intragranular α. Meanwhile, such minor increase of inter-particle spacing of intragranular α resulted in minor increase of slip distance for dislocation movement, and exhibited the limited improvement of ductility. This trend of strength and ductility variation also occurred in other near β titanium alloys, such as Ti-3.5Al-5Mo-6V-3Cr-2Sn-0.5Fe (wt.%) alloy and Ti-3.5Al-5Mo-4V-2Cr-1Fe-2Zr -2Sn-0.1B (wt.%) alloy [38,39]. When aged temperature reached as high as 600 °C, the strength decreased significantly and the averaged width and inter-particle spacing of intragranular α increased greatly. It can be deduced that the alloy strength is mainly determined by size and quantity of intragranular α. However, because of the formation of $\alpha_{\mathrm{WGB}}$, the crack occurred not only at grain boundary α/β interface but also at $\alpha_{\mathrm{WGB}}$/β interface. Therefore, the $\alpha_{\mathrm{WGB}}$ deflected the crack propagation direction and resulted in the improvement of ductility. In addition, the broad inter-particle spacing of intragranular α further enhanced the ductility. According to Figure 10c,d, with the increase of aging time, minor change in tensile properties could be related to the minor variation of averaged width and inter-particle spacing of intragranular α.

## 5. Conclusions

A near β titanium alloy Ti-6Mo-5V-3Al-2Fe was newly developed. The evolution of secondary α phase influenced by aging temperature and aging time was investigated; the relationship between secondary α phase on mechanical properties was discussed. The following conclusions can be drawn from the present investigation.

(1) The athermal ω phase formed after water quenching and disappeared after aged at 600 °C. The ω phase assisted the nucleation of intragranular α phase during aging treatment.
(2) When aged at 480 °C, fine intragranular α precipitated because of the synergetic contribution of ω-assisted nucleation, high nucleation rate and slow growth rate. The width and inter-particle spacing of intragranular α increased slightly as the aging temperature increased from 480 °C to 560 °C, but increased considerably when the aging temperature reached as high as 600 °C.
(3) When aged at 600 °C, the size and quantity of intragranular α were not sensitive to the aging time ranging from 4 h to 16 h. After aged at 600 °C, the mutually parallel $\alpha_{\mathrm{WGB}}$ formed on grain boundary α and grew into the internal areas of β grains.
(3) The alloy aged at 480 °C exhibited high strength but poor ductility, with the UTS and EL are 1510 MPa and 4.2%, respectively. As the aging temperature increased to 560 °C, the minor variation of intragranular α led to slight decrease of strength and minor improvement of ductility. When aging temperature reached 600 °C, the strength decreased seriously but ductility improved dramatically, resulting from boarder inter-particle spacing of intragranular α and the formation of $\alpha_{\mathrm{WGB}}$.

## Figures and Tables

**Table 1:** Averaged width and inter-particle spacing of intragranular α after aging treatments.

Aging Treatment	480 °C/4 h	520 °C/4 h	560 °C/4 h	600 °C/4 h	600 °C/8 h	600 °C/16 h
**Averaged width (nm)**	67	93	124	231	257	287
**Averaged inter-particle spacing (nm)**	90	131	142	246	282	290

## References

[1] Zhao J., Zhong J., Yan F., Chai F., Dargusch M. (2017). Deformation Behaviour and Mechanisms during Hot Compression at Supertransus Temperatures in Ti-10V-2Fe-3Al. J. Alloys Compd..

[2] Fan X.G., Zhang Y., Gao P.F., Lei Z.N., Zhan M. (2017). Deformation Behavior and Microstructure Evolution during Hot Working of A Coarse-grained Ti-5Al-5Mo-5V-3Cr-1Zr Titanium Alloy in Beta Phase Field. Mater. Sci. Eng. A.

[3] Zhang J., Wang Y., Zhang B., Huang H.J., Chen J.H., Wang P. (2018). Strain Rate Sensitivity of Tensile Properties in Ti-6.6Al-3.3Mo-1.8Zr-0.29Si Alloy: Experiments and Constitutive Modeling. Materials.

[4] Liu X., Yu D.H., Fan Q.B., Shi R. (2017). Influence of Hot Rolling and Heat Treatment on the Microstructural Evolution of β20C Titanium Alloy. Materials.

[5] Lhadi S., Chini M.R., Richeton T., Gey N., Germain L., Berbenni S. (2018). Micromechanical Modeling of the Elasto-Viscoplastic Behavior and Incompatibility Stresses of β-Ti Alloys. Materials.

[6] Shekhar S., Sarkar R., Kar S.K., Bhattacharjee A. (2015). Effect of Solution Treatment and Aging on Microstructure and Tensile Properties of High Strength β Titanium Alloy, Ti–5Al–5V–5Mo–3Cr. Mater. Des..

[7] Du Z.X., Xiao S.L., Xu L.J., Tian J., Kong F.T., Chen Y.Y. (2014). Effect of Heat Treatment on Microstructure and Mechanical Properties of a New β High Strength Titanium Alloy. Mater. Des..

[8] Ivasishin O.M., Markovsky P.E., Semiatin S.L., Ward C.H. (2005). Aging Response of Coarse- and Fine-grained Titanium Alloys. Mater. Sci. Eng. A.

[9] Angelier C., Bein S., Béchet J. (1997). Building a Continuous Cooling Transformation Diagram of β-CEZ Alloy by Metallography and Electrical Resistivity Measurements. Metall. Mater. Trans. A.

[10] Ivasishin O.M., Markovsky P.E., Matviychuk Y.V., Semiatin S.L., Ward C.H., Fox S. (2008). A Comparative Study of the Mechanical Properties of High-strength β-titanium Alloys. J. Alloys Compd..

[11] Ma X.K., Li F.G., Cao J., Li J.H., Sun Z.K., Zhu G., Zhou S.S. (2018). Strain Rate Effects on Tensile Deformation Behaviors of Ti-10V-2Fe-3Al Alloy Undergoing Stress-induced Martensitic Transformation. Mater. Sci. Eng. A.

[12] Huang L.G., Chen Y.Y. (2015). A Study on the Microstructures and Mechanical Properties of Ti–B20–0.1B Alloys of Direct Rolling in the α + β Phase Region. J. Alloys Compd..

[13] Li C., Chen J., Li W., Ren Y.J., He J.J., Song Z.X. (2016). Effect of Heat Treatment Variations on the Microstructure Evolution and Mechanical Properties in a β Metastable Ti Alloy. J. Alloys Compd..

[14] Ren L., Xiao W.L., Chang H., Zhao Y.Q., Ma C.L., Zhou L. (2018). Microstructural Tailoring and Mechanical Properties of a Multi-alloyed Near β Titanium Alloy Ti-5321 with Various Heat Treatment. Mater. Sci. Eng. A.

[15] He T., Feng Y., Luo W.Z., He Y.S., Tian L., Lai Y.J. (2018). Microstructural Evolution of ω Assisted α Precipitates in β-CEZ Alloy during Ageing Process. Mater. Charact..

[16] Ahmed M., Wexler D., Casillas G., Ivasishin O.M., Pereloma E.V. (2015). The Influence of β Phase Stability on Deformation Mode and Compressive Mechanical Properties of Ti–10V–3Fe–3Al Alloy. Acta. Mater..

[17] Hua K., Zhang Y.D., Kou H.C., Li J.S., Gan W.M., Fundenberger J.J., Esling C. (2017). Composite Structure of α Phase in Metastable β Ti Alloys Induced by Lattice Strain during β to α Phase Transformation. Acta. Mater..

[18] Abdel-Hady M., Hinoshita K., Morinaga M. (2006). General Approach to Phase Stability and Elastic Properties of β-type Ti-alloys Using Electronic Parameters. Scr. Mater..

[19] Brozek C., Sun F., Vermaut P., Millet Y., Lenain A., Embury D., Jacques P.J., Prima F. (2016). A β-titanium Alloy with Extra High Strain-hardening Rate: Design and Mechanical Properties. Scr. Mater..

[20] Ren L., Xiao W.L., Ma C.L., Zheng R.X., Zhou L. (2018). Development of a High Strength and High Ductility Near β-Ti alloy with Twinning Induced Plasticity Effect. Scr. Mater..

[21] Kuroda D., Niinomi M., Morinaga M., Kato Y., Yashiro T. (1998). Design and mechanical properties of new β type titanium alloys for implant materials. Mater. Sci. Eng. A.

[22] Cotton J.D., Briggs R.D., Boyer R.R., Tamirisakandala S., Russo P., Shchetnikov N., Fanning J.C. (2015). State of the Art in Beta Titanium Alloys for Airframe Applications. JOM.

[23] Wang K., Li M.Q. (2014). Effects of Heat Treatment and Hot Deformation on the Secondary α Phase Evolution of TC8 Titanium Alloy. Mater. Sci. Eng. A.

[24] Nag S., Banerjee R., Srinivasan R., Hwang J.Y., Harper M., Fraser H.L. (2009). ω-Assisted Nucleation and Growth of α Precipitates in the Ti–5Al–5Mo–5V–3Cr–0.5Fe β Titanium Alloy. Acta Mater..

[25] Li C.L., Mi X.J., Ye W.J., Hui S.X., Lee D.G., Lee Y.T. (2015). Microstructural Evolution and Age Hardening Behavior of a New Metastable Beta Ti–2Al–9.2Mo–2Fe Alloy. Mater. Sci. Eng. A.

[26] Xu T.W., Zhang S.S., Zhang F.S., Kou H.C., Li J.S. (2016). Effect of ω-assisted Precipitation on β→α Transformation and Tensile Properties of Ti–15Mo–2.7Nb–3Al–0.2Si Alloy. Mater. Sci. Eng. A.

[27] Zheng Y.F., Williams R., Wang D., Shi R.P., Nag S., Kami P., Sosa J.M., Banerjee R., Wang Y.Z., Fraser H.L. (2016). Role of ω Phase in the Formation of Extremely Refined Intragranular α Precipitates in Metastable β-titanium Alloys. Acta Mater..

[28] Prima F., Vermaut P., Texier G., Ansel D., Gloriant T. (2006). Evidence of α-nanophase Heterogeneous Nucleation From ω Particles in a β-metastable Ti-based Alloy by Highresolution Electron Microscopy. Scr. Mater..

[29] Yi R.W., Liu H.Q., Yi D.Q., Wan W.F., Wang B., Jiang Y., Yang Q., Wang D.C., Gao Q., Xu Y.F. (2016). Precipitation hardening and microstructure evolution of the Ti–7Nb–10Mo alloy during aging. Mater. Sci. Eng. C.

[30] Dong R.F., Li J.S., Fan J.K., Kou H.C., Tang B. (2017). Precipitation of α Phase and Its Morphological Evolution during Continuous Heating in a Near β Titanium Alloy Ti-7333. Mater. Charact..

[31] Zhang X., Kou H.C., Li J.S., Zhang F.S., Zhou L. (2013). Evolution of the Secondary a Phase Morphologies during Isothermal Heat Treatment in Ti-7333 Alloy. J. Alloys Compd..

[32] Xavier C.C., Correa D.R.N., Grandini C.R., Rocha L.A. (2017). Low Temperature Heat Treatments on Ti-15Zr-xMo Alloys. J. Alloys Compd..

[33] Fan J.K., Li J.S., Kou H.C., Hua K., Tang B., Zhang Y.D. (2016). Microstructure and mechanical property correlation and property optimization of a near b titanium alloy Ti-7333. J. Alloys Compd..

[34] Nag S., Banerjee R., Hwang J.Y., Harper M., Fraser H.L. (2009). Elemental Partitioning Between α and β Phases in the Ti–5Al–5Mo–5V–3Cr–0.5Fe (Ti-5553) Alloy. Philos. Mag..

[35] Zheng Y.F., Choudhuri D., Alam T., Williams R.E.A., Banerjee R., Fraser H.L. (2016). The Role of Cuboidal ω Precipitates on α Precipitation in a Ti-20V Alloy. Scr. Mater..

[36] Guo S., Zhang J.S., Cheng X.N., Zhao X.Q. (2015). A Metastable β-type Ti-Nb Binary Alloy with Low Modulus and High Strength. J. Alloys Compd..

[37] Devaraj A., Joshi V.V., Srivastava A., Manandhar S., Moxson V., Duz V.A., Lavender C. (2016). A low-cost hierarchical nanostructured beta-titanium alloy with high strength. Nat. Commun..

[38] Chen Y.Y., Du Z.X., Xiao S.L., Xu L.J., Tian J. (2014). Effect of aging heat treatment on microstructure and tensile properties of a new β high strength titanium alloy. J. Alloys Compd..

[39] Huang L.G., Chen Y.Y. (2015). A study on the microstructures and mechanical properties of forged trace-boron-modified Ti–B20 alloy. Mater. Des..

